# Towards better estimates of uncorrected presbyopia

**DOI:** 10.2471/BLT.15.156844

**Published:** 2015-10-01

**Authors:** Brien A Holden, Nina Tahhan, Monica Jong, David A Wilson, Timothy R Fricke, Rupert Bourne, Serge Resnikoff

**Affiliations:** a Deceased, formerly Brien Holden Vision Institute, Sydney NSW 2052, Australia.; b Brien Holden Vision Institute, Level 4 North Wing, Rupert Myers Building, Gate 14 Barker Street, University of New South Wales, Sydney NSW 2052, Australia.; c Vision and Eye Research Unit, Anglia Ruskin University, Cambridge, England.

Normal vision depends upon the ability of the ocular lens to change shape, ensuring that light is focused on the most sensitive part of the retina. Anyone living beyond middle age is inevitably affected by presbyopia, an inability to focus on near objects, due to the loss of flexibility of the ocular lens. It is estimated that over half of the one billion people affected globally cannot afford the spectacles needed to correct their eyesight. Most of the people with uncorrected visual impairment live in low- and middle-income countries.^1^^,^^2^

In 2002, the World Health Organization (WHO) estimated that more than 161 million people had visual impairment and that cataract was the leading cause.^3^ However, at that time, vision impairment that could be prevented “with use of the best possible optical correction” was excluded from the category H54 (visual impairment including blindness) in WHO’s *International statistical classification of diseases and related health problems* (ICD).^4^ Various groups with expertise on vision impairment argued that this definition was meaningless if no optical correction were available.^5^ Revised estimates of the number of people with vision impairment, including uncorrected refractive error, were published in 2004, 2010 and 2013.^6^^–^^8^ Unfortunately, even in these revised estimates, impairment of near vision was not included due to insufficient data on the prevalence of the condition.

Research that has specifically set out to investigate the prevalence and impact of uncorrected refractive error has often only considered impairment of distance vision.^9^ Among people who would benefit from the use of spectacles, it is estimated that 108 million people worldwide have impaired distance vision,^8^ while nearly five times as many (517 million people) have impaired near vision.^1^

Near vision is often perceived to be less problematic than impaired distance vision, particularly for people in low- and middle-income countries. It could be argued that reduced literacy, differences in vocation and lower life expectancy reduce the need for near vision correction – but recent studies indicate the contrary. Studies in rural Africa have shown that near vision impairment greatly impacts quality of life despite very low literacy levels.^10^ In these settings, spectacles have been found to be essential for a range of activities including sorting grains, weeding, cooking, sewing and caring for children.^10^ Illiterate participants provided with spectacles were found to be just as likely to recommend spectacles as literate participants.^11^ Impairment of near vision is at least as detrimental to quality of life as impairment of distance vision, regardless of the setting, sociodemographics or lifestyle of participants.^12^

Uncorrected presbyopia is the most common cause of visual impairment. WHO has recommended the measurement of near vision in population-based surveys. Formal inclusion of near vision impairment in the ICD is an overdue and crucial step in dealing effectively with this common but easily mitigated disability.
